# Prevalence of needle stick and its related factors in Iranian health worker: an updated systematic review and meta-analysis

**DOI:** 10.7189/jogh.13.04104

**Published:** 2023-10-02

**Authors:** Hadis Fathizadeh, Zahra Alirezaie, Fatemeh Saeed, Bita Saeed, Zahra Gharibi, Abdol R Biojmajd

**Affiliations:** 1 Department of Laboratory Sciences, Sirjan School of Medical Sciences, Sirjan, Iran; 2 BS in Nursing, Baft Khatam Ol-Anbia Hospital, Kerman University of Medical Sciences, Kerman, Iran; 3 Student Research Committee, Sirjan School of Medical Sciences, Sirjan, Iran; 4 Infectious and Tropical Diseases Research Center, Hormozgan Health Institute, Hormozgan University of Medical Sciences, Bandar Abbas, Iran

## Abstract

**Background:**

Healthcare workers (HCWs) are at risk of acquiring blood-borne infections such as hepatitis B, hepatitis C, and human immunodeficiency virus through needlestick injuries (NSIs). We aimed to investigate the prevalence of needlestick injuries and other related indicators among HCWs in Iran through a systematic review and meta-analysis.

**Methods:**

We searched various databases until the end of May 2023 for studies reporting the prevalence of NSIs among healthcare workers in Iran. We used a random model with 95% confidence intervals (CIs) to analyse the data and the Joanna Briggs Institute (JBI) tool to evaluate the quality of included studies. We conducted and reported the study according to the Preferred Reporting Items for Systematic Reviews and Meta-Analyses (PRISMA) statement.

**Results:**

We included 87 studies in the analysis and found that 47% (95% CI = 42-52, *I*^2^ = 98.9%) of Iranian HCWs experienced NSI. NSIs were most frequently related to syringe needles (58%; 95% CI = 52-65, *I*^2^ = 96.8%) and most often caused by recapping (30%; 95% CI = 22-38, I^2^ = 98.5%). In this study, 56% (95% CI = 45-67, *I*^2^ = 98.6%) of HCWs with NSIs did not report their injury. Moreover, the prevalence of NSIs the highest in the morning shift (0.44; 95% CI = 0.36-0.53, *I*^2^ = 97.2%), emergency unit (0.20; 95% CI = 0.16-0.24, *I*^2^ = 93.7%), and intensive care unit (0.20; 95% CI = 0.16-0.24, *I*^2^ = 94.3%).

**Conclusions:**

To reduce the high prevalence of NSIs, HCWs, especially those in emergency departments, should use safety equipment. Healthcare managers should provide a calm and stress-free environment for HCWs, educate them on safety principles and standards, and support experienced HCWs with NSIs.

Needlestick injuries (NSIs) are one of the most common occupational injuries and errors among health care workers (HCWs) worldwide [1], causing blood-borne transmission of various pathogens [2,3]. Studies have found that more than 20 other infections, such as hepatitis B (HBV), hepatitis C (HCV), human immunodeficiency virus (HIV), syphilis, tetanus, malaria, and tuberculosis, can be transmitted through non-surgical sharp injuries, highlighting the importance of proper precautions in preventing such transmissions [4-6]. Of these diseases, HBV, HCV, and HIV are the most significant, as reports have suggested that over three million HCWs annually are at risk of exposure due to sharp injuries. A previous study found that the risk of disease transmission through NSIs was 0.2-0.5% for HIV, 3-10% for HCV, and 40% for HBV [7]. Thus, implementing effective preventive measures to minimise the risk of such incidents is crucial [8]. 

Every non-surgical sharp injury incurs both direct and indirect costs on the healthcare system, ranging from US$175 to US$350. Therefore, reducing the incidence of NSIs should be a priority for healthcare institutions to minimise financial burdens and improve patient care [9]. However, the treatment of transmitted diseases and absenteeism also brings high costs to healthcare systems [10]. Despite efforts by many governments to mitigate the occurrence of NSIs, such incidents continue to happen frequently, resulting in significant financial burdens [11].

Injuries mostly happen during needle recapping, operative procedures, blood sample collection, intravenous (IV) line administration, and poor waste disposal practices [6]. Furthermore, NSIs are associated with several different factors beyond the control of health workers, including excessive workload, working in an intensive care unit (ICU), being female, lacking job experience, and being young [12].

Different studies have indicated that the occurrence of NSIs is 54.6% in the United States [12], 40.2% in Nigeria [13], and 9.7% in Switzerland [14]. Due to their significance, reporting NSIs to advance prevention and treatment remains essential, yet only 10% of HCWs who suffered from an NSI reported on the incident, as per the report released by Iran's Center for Disease Control and Prevention [15,16].

Systematic reviews and meta-analyses conducted in Iran have not examined individual factors influencing NSIs and their characteristics among HCWs [17-19]. To address this gap, we aimed to investigate the prevalence of NSIs and the associated factors contributing to the occurrence of such injuries among HCWs, in order to advance the understanding of NSIs and suggest improvements for interventions to prevent such incidents.

## METHODS

We conducted a systematic review and meta-analysis to investigate the risk factors of NSIs among HCWs in Iran until May 2023, according to the Preferred Reporting Items for Systematic Reviews and Meta-Analysis (PRISMA) statement [20]. The Research Ethics Committee of Sirjan Faculty of Medical Sciences (IR.SIRUMS.REC.1402.004) approved this study. 

### Search strategy and selection criteria

Two researchers (ABB, HF) searched Scopus, PubMed, Web of Science, Google Scholar, Science Direct, SID, and MagIran using relevant keywords (e.g. “needle stick,” “sharp injury,” “needle* stick injuries*,” “injur*,” “needlestick injur*,” “sharp*,” and “Iran”). Two researchers (ARB and HF) independently reviewed the titles and abstracts, followed by the full texts of the retrieved studies. We also manually searched the references of the included studies. We contacted authors in cases where full texts of studies were unavailable.

We included cross-sectional studies that reported the prevalence of NSIs among HCWs over a lifetime or a period of time, limited to studies in Persian and English languages. We excluded studies on students, dentists, and home care workers (due to the lack of organised reporting of needlestick injuries in Iran for this group of HCWs), and all secondary and elementary studies (except for descriptive studies).

### Data extraction and quality assessment

Two researchers (ABB and ZA) independently evaluated each article based on pre-defined inclusion criteria. Using a Microsoft Excel sheet (Microsoft Corporation, Redmond, Washington, USA), two researchers (ABB and BS) independently extracted the following data from included studies: sample size, prevalence of NSIs, demographic characteristics of participants, publication date, factors associated with NSIs, and study location.

Two researchers (ABB and FS) used the critical appraisal checklist developed by the Joanna Briggs Institute (JBI) to evaluate the quality of the studies included in our research. This checklist is specifically designed for cross-sectional studies and comprises eight domains. Studies can be categorised as high (>6), moderate (3-6) or low quality (<3). We did not exclude any studies based on their quality score (Tables S1 and S2 in the **Online Supplementary Document**). This assessment was done twice. In case of uncertainty or disagreement between the two researchers, an independent investigator was consulted for resolution (HF).

### Statistical analysis

We considered NSIs as the outcome variable and other ones, such as shift work, department of service, etc., as independent variables affecting NSIs. We combined the prevalence rates of different studies using a weighted average method, where each study’s weight was determined by the inverse of its variance. We used the *I*^2^ statistical measure for the degree of heterogeneity to assess the heterogeneity of data, categorised as low heterogeneity (*I*^2^ <25%), average heterogeneity (*I*^2^ = 25-75%), and high heterogeneity (*I*^2^ >75%). In this study, we analysed the data using a random effects model due to the high heterogeneity (*I*^2^>75%). We conducted subgroup analyses to evaluate the prevalence of NSIs based on factors such as shift work, injurious devices, cause of NSI, hospital ward, and others. We employed sensitivity analyses to investigate the impact of individual studies on the final results and used Egger's and Begg's regression tests to assess publication bias and the influence of small studies. We used Stata, version 17.0 (StataCorp LLC, College Station, Texas, USA) for data analysis.

## RESULTS

**Figure 1:**
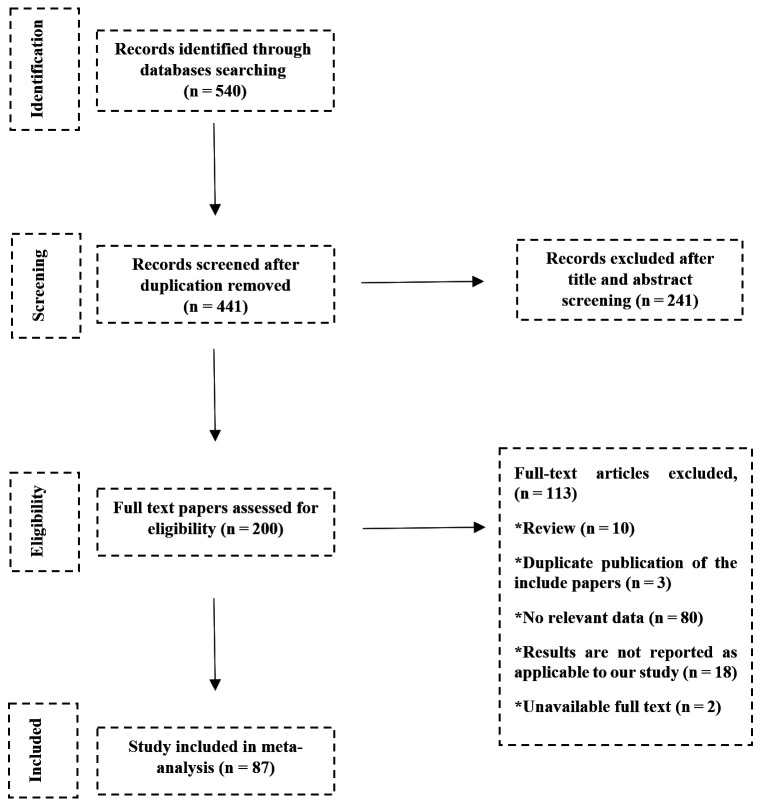
The PRISMA flow diagram showing the study selection process.

We retrieved 540 studies from the database search; 453 remained after deduplication. Two hundred studies remained for full-text evaluation following title and abstract screening. Finally, we included 87 studies in our final analysis (**Figure 1**) on 29 815 participants, with an average of 353 individuals per study. Askarian et al. [21] had the largest sample size with 1555 participants, while Mohammadi-Najafabadi et al. [22] and Hajivandi et al. [23] had the smallest, with 68 participants each (Table S3 in the **Online Supplementary Document** and **Table 1**).

**Table 1:** Effective subgroups in the needle stick of Iranian health workers

Variable	Sample size	ES (95% CI)	I^2^
Shift work			
*Morning*	21	0.44 (0.36-0.53)	97.2
*Evening*	15	0.19 (0.14-0.25)	95.7
*Night*	17	0.35 (0.23-0.47)	98.7
Injurious device			
*Angio catheter*	36	0.25 (0.19-0.31)	98.1
*Syringe needle*	38	0.58 (0.52-0.65)	96.8
*Suture needle*	28	0.12 (0.09-0.15)	94.5
Cause of NSI			
*Recapping*	35	0.30 (0.22-0.38)	98.5
*Veins*	22	0.26 (0.22-0.31)	87.5
*IV*	21	0.18 (0.13-0.23)	94.3
*IM-S.C*	11	0.14 (0.10-0.19)	84.5
Hospital ward			
*Emergency*	23	0.20 (0.16-0.24)	93.7
*Internal*	16	0.18 (0.12-0.23)	95.0
*Surgery*	13	0.19 (0.14-0.23)	89.2
*ICU and CCU dialysis*	22	0.20 (0.16-0.24)	94.3
Psychiatry	5	0.02 (0.01-0.04)	34.5
Cause of non-reporting			
*High busy*	8	0.24 (0.16-0.32)	89.0
*lack awareness*	6	0.18 (0.12-0.23)	65.5
*Dissatisfaction*	7	0.22 (0.14-0.30)	93.0
*Low risk*	8	0.26 (0.12-0.40)	97.5
Event due to injury			
*high volume of work*	17	0.43 (0.36-0.50)	93.6
*Haste*	10	0.25 (0.19-0.30)	83.6
*Colleague*	7	0.11 (0.07-0.15)	77.3
*Patient*	8	0.17 (0.11-0.23)	90.5
Target personnel			
*Nurse*	38	0.51 (0.44-0.57)	98.3
*Health worker*	45	0.43 (0.37-0.49)	98.9
Actions after NSI			
*Washing*	20	0.48 (0.36-0.59)	98.8
*Pressing to remove blood*	11	0.26 (0.14-0.39)	98.7
*No action*	6	0.02 (0.01-0.03)	44.4

ES – effect size, CI – confidence interval, NSI – needlestick injuries, IV – intravenous, IM-SC – intramuscular-subcutaneous, CCU and ICU – coronary care unit and intensive care unit

**Figure 2:**
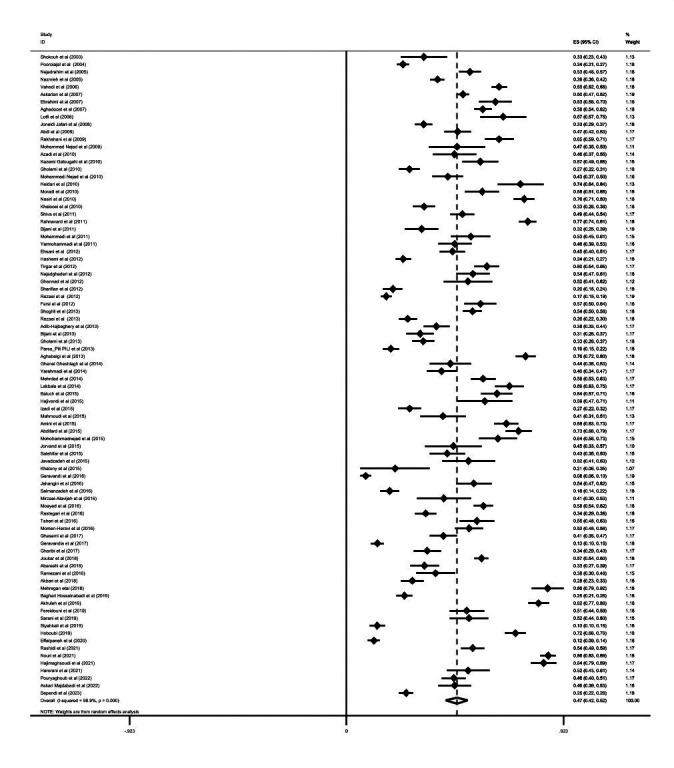
Frequency of needlestick injuries among Iranian health workers in the present study. ES – effect size, CI – confidence interval.

The reported prevalence of NSIs in all studies ranged from 8% to 86% (**Figure 2**). According to the results of the random effects method, the prevalence of NSIs across all studies was 47% (95% CI = 42-52; *I*^2^ = 98.9%). The prevalence rates of NSIs in nurses and health workers were about 0.51 (95% CI = 0.42-0.57) and 0.43 (95% CI = 0.37-0.49), respectively (**Table 1**). We found the prevalence of NSIs to be higher for morning shifts (0.44; 95% CI = 0.36- 0.53, *I*^2^ = 97.2%) compared to all other shift work. The prevalence of NSIs among HCWs was highest in emergency units and coronary care unit (CCU) and ICU dialysis (0.20; 95% CI = 0.16-0.24; I^2^ = 93.7% and I^2^ = 94.3%)

**Figure 3:**
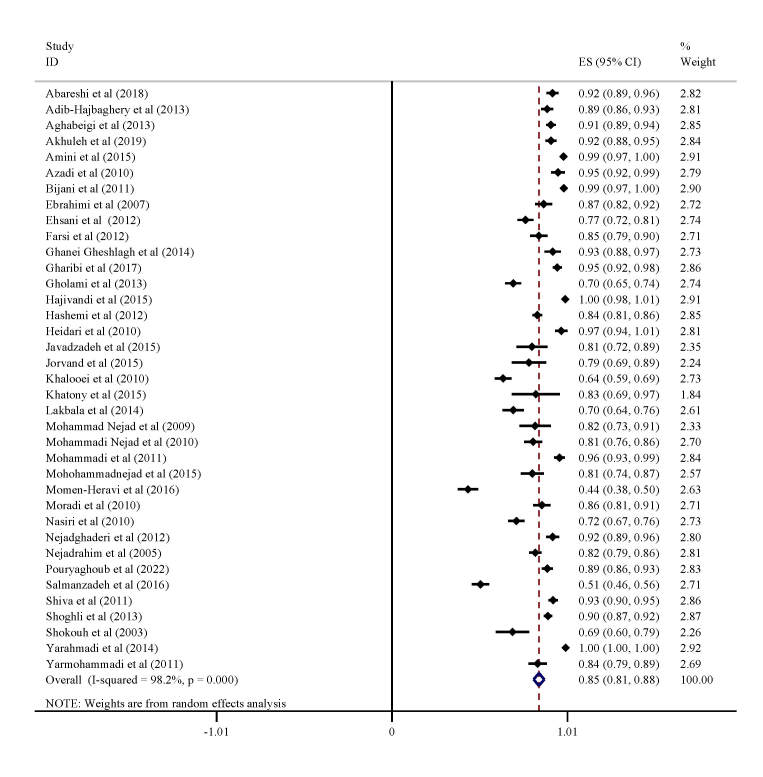
Vaccination status against hepatitis B in Iranian health workers. ES – effect size, CI – confidence interval.

The prevalence of complete vaccination against hepatitis B was 44-100% (**Figure 3**) in all studies, while the random effects method estimated it to be 85% (95% CI = 81-88, I^2^ = 98.2%).

**Figure 4:**
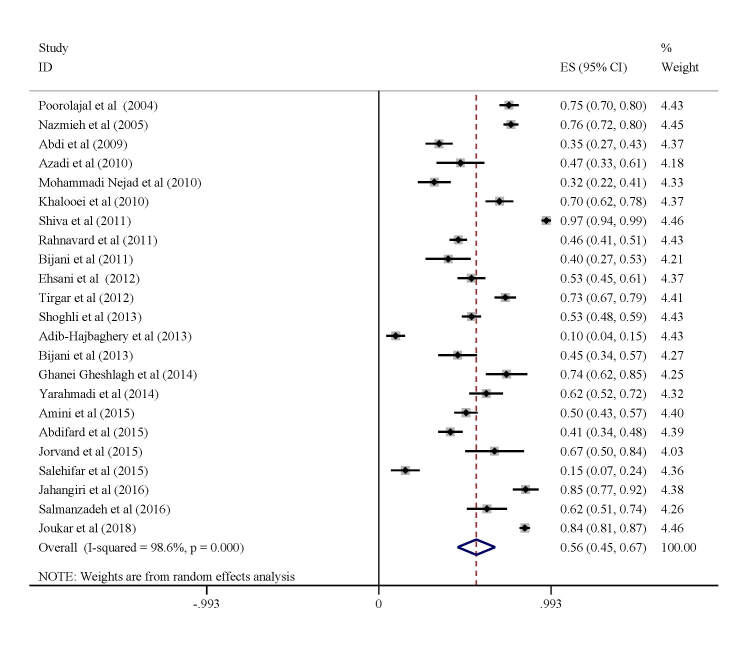
Failure to report needle stick injuries in injured healthcare workers. ES – effect size, CI – confidence interval.

Due to the high heterogeneity among the selected studies, we used a random-effects model to estimate the overall prevalence of non-reporting of NSIs to nursing managers among nurses in Iran. The model estimated a 56% (95% CI = 45-67, I^2^ = 98.6%) total incidence rate of unreported NSIs during the study period (**Figure 4**). The most frequent reasons for not reporting NSIs were low risk of disease transmission (26%; 95% CI = 12-40, *I*^2^ = 97.5%) and high workload (24%; 95% CI = 16-32, *I*^2^ = 89.0%) (**Table 1**).

Derived from the outcomes obtained via the random effects approach, NSIs were most frequently caused by syringe needle (58%; 95% CI = 52-65, I^2^ = 96.8%) compared to other medical devices, while recapping (0.30%; 95% CI = 0.22-0.38) was the most reported activity leading to NSIs. Additionally, the most frequent measures taken following a NSI were washing the affected area with soap and water.

**Figure 5:**
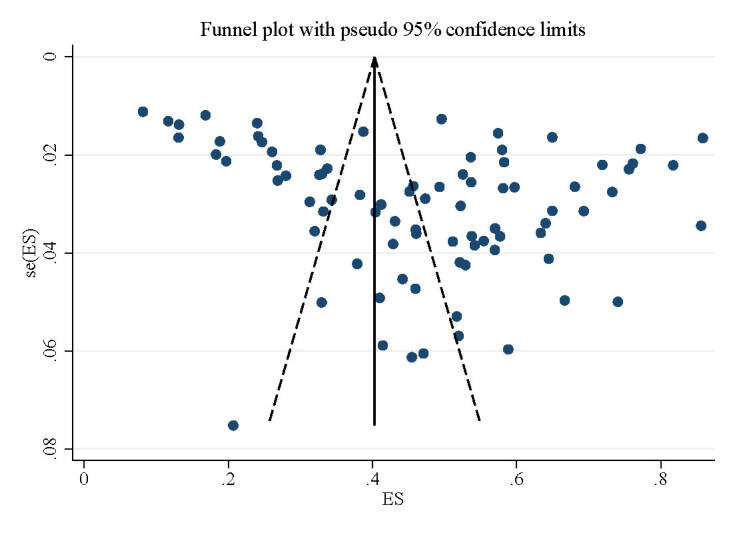
Funnel plot for assessing the risk of publication bias.

The sensitivity analysis showed that no solitary study had a significant effect on the prevalence of NSIs. To assess publication bias, we employed funnel plots, and Begg and Egger's tests. Each dot on the funnel plot represents a distinct study, and an uneven distribution provides evidence of publication bias [24,25]. We plotted the study effect sizes against their standard errors and evaluated the ensuing funnel plots, which indicated publication bias for the prevalence of NSIs due to asymmetry (**Figure 5**). Although the Begg test results (*P* = 0.214) did not indicate any signs of publication bias, the outcomes of the Egger test (*P* = 0.00) suggested otherwise.

## DISCUSSION

NSI is one of the important health risks that medical and health workers face in the medical environment. We conducted a systematic review and meta-analysis and found that nearly 50% of the participants had incurred injuries from sharp instruments. We determined that the prevalence of NSIs among healthcare workers in Iran was 47% (95% CI = 42-51), consistent with the results of previous studies by Alimohamadi et al. (50.8%) [11], Rezai et al. (42.0%) [18], and Ghanei Gheshlagh et al. (42.5%) [26], but higher than the study in Ethiopia by Yazie et al. (28.8%) [27]. Possible reasons for the variations between our study and that of Yazei et al. [27] may be the different inclusion periods, countries, or sample sizes. The prevalence of NSIs varies depending on gender, age, work experience, hospital ward, and skills [28]. Through educational programs, HCWs can acquire the necessary knowledge and skills for effectively managing needles and other sharp medical instruments, including the correct methods of handling, holding, and disposing of needles, as well as understanding the potential risks associated with NSIs. Moreover, education can provide information about the significance of employing personal protective equipment like gloves, masks, and goggles, which serve as an added layer of protection [29,30].

In our study and other previous ones, NSIs were more common in nurses (0.51; 95% CI = 0.44-0.57) than in other HCWs (0.43; 95% CI = 0.37-0.49) [11,31]. Yoshika et al. [32] reported NSI injuries were more frequent in nurses than in doctors in Japan due to the difference in their work in hospitals. Nurses are at greater risk than other HCWs, as they more frequently work with needles and other sharp instruments. They perform procedures such as blood sampling, intravenous, intramuscular, subcutaneous injections, or intradermal injections, and sutures, which increases the risk of needle stick [11,16,33,34].

Recapping (0.30%; 95% CI = 0.22-0.38) was the most reported activity leading to NSIs, which is in line with previous [35-38]. This may be related to a lack of knowledge, lack of needle-crushing machines, mandatory hospital instructions, and the HCWs’ high workload [39]. Syringes with needles 0.58% (95% CI = 0.52-0.65) and angio-catheter (0.25%; 95% CI = 0.19-0.31) were objects most frequently causing NSIs, which could be attributed to their frequent utilisation in Iran, combined with insufficient training [12]. This finding was consistent with several previous studies [40-42]. Izadi et al. [43] identified blood drawing as the primary cause of NSIs among HCWs. Factors such as inadequate experience in phlebotomy and the condition of the veins can also play a significant role in the occurrence of this problem. To minimise the occurrence of NSIs, it is crucial to design and implement safety devices which incorporate various protective mechanisms to facilitate easier access to veins and reduce the likelihood of accidental NSIs. For instance, one commonly used invasive therapeutic approach in hospitals is the utilisation of angio-catheters to access the venous network. These devices play a significant role in reducing errors and consequently decreasing the occurrence of NSIs among HCWs.

We found that two most prevalent measures taken following a NSIs were applying pressure to the affected region (26%) and washing the area with soap and water (48%), which aligns with studies by Joukar et al. and Adib-Hajbaghery et al. [2,44]. However, some studies have reported that a number of health care workers do not take any action after a NSI [45]. These findings indicate a lack of knowledge and attitude among health care workers regarding the risks of NSIs, highlighting a need for training in this area. Practical training on NSIs cannot only prevent such incidents, but encourages appropriate actions to be taken even after an injury occurs, as well as proper follow-up measures [46].

We determined that 85% (95% CI = 81-88) of HCWs had been administered the hepatitis B (HBV) vaccine. These findings are consistent with previous studies conducted in Iran [12,47]. According to the study by Burnett et al. [48] conducted in Africa, only 19.9% of HCWs had received complete vaccination. The variations observed might be due to the usage of diverse protocols for administering vaccinations to health care workers (HCWs) in hospitals. However, receiving the hepatitis vaccine may have led to false assurance and carelessness in HCWs.

According to our findings, 56% (95% CI = 45-67) of HCWs did not report their NSIs. Meanwhile, Laishram et al. [49] estimated non-reporting of NSI to be at 43% among HCWs, while another systematic review and meta-analysis reported that more than half of HCWs in Iran do not report NSIs [15]. Reporting injury leads to post-exposure prophylaxis, early detection of probable infection, and the provision of effective treatment. In this study, the low risk of disease transmission was an important reason for the non-reporting of NSIs, which is consistent with the results of previous studies [50]. The inadequate reporting of NSIs by HCWs could be correlated with the absence of a transparent reporting protocol.

HCWs in emergency departments and intensive care units were more likely to experience NSIs compared to those in other departments, possibly due to their emergent nature and stressful environment [35]. The high incidence of NSIs among emergency nurses can be attributed to factors such as the dynamic and fast-paced nature of emergency care, the increased likelihood of encountering patients with infectious diseases, the frequent use of needles and sharp instruments, and the high-pressure conditions in emergency settings. Similar to previous research, the NSI incidents were most prevalent during the morning shift (0.44%; 95% CI = 0.36-0.53), which could be due to the increased number of patients being attended during those hours [51-53].

This study has some limitations. We only included studies on Iranian HCWs, and the limitation of their cross-sectional should be considered when interpreting our findings. Additionally, data in most studies were collected based on self-reporting, which may affect the prevalence of needle-stick injuries. We were also unable to investigate other aspects of NSIs in Iran, such as gender, education level, and prevalence of needle-stick incidents.

## CONCLUSIONS

We found that approximately half of the HCWs in the included studies experienced NSIs. To reduce these injuries, hospital managers should assess needle-stick-related factors, provide safety devices, and ensure a calm and stress-free environment for staff, while advocating for safety principles to young HCWs and supporting HCWS who are injured to create a culture of reporting NSIs.

## Additional material


Online Supplementary Document
