# Supplementary material for: Prevalence of needle stick and its related factors in Iranian health worker: an updated systematic review and meta-analysis
Source: J Glob Health. 2023 Oct 2;13:04104. doi: 10.7189/jogh.13.04104 (PMC10543000; doi:10.7189/jogh.13.04104)
Supplement: Online Supplementary Document [file jogh-13-04104-s001.pdf]

Prevalence of needle stick and its related factors in Iranian health worker: an updated systematic review and meta-analysis

Running title: Fathizadeh et al. Prevalence of needle stick in Iranian health workers

Hadis Fathizadeh[1](https://orcid.org/0000-0003-2248-6665), Zahra Alirezaie[2](https://orcid.org/0000-0002-4060-0073), Fatemeh Saeed[3](https://orcid.org/0000-0001-6283-1203), Bita Saeed[3](https://orcid.org/0000-0001-5179-403X), Zahra Gharibi[4](https://orcid.org/0000-0001-7462-122X), Abdol rahim biojmajd[3](https://orcid.org/0000-0002-8192-1180)

1- Department of laboratory sciences, Sirjan faculty of medical sciences, Sirjan, Iran.

2- BS in Nursing, Baft Khatam Ol-Anbia Hospital, Kerman University of Medical Sciences, Kerman, Iran.

3- Student Research Committee, Sirjan School of Medical Sciences, Sirjan, Iran.

4- Infectious and Tropical Diseases Research Center, Hormozgan Health Institute, Hormozgan University of Medical Sciences, Bandar Abbas, Iran

First author	Q1	Q2	Q3	Q4	Q5	Q6	Q7	Q8	Quality
Shokouh (1)	N	N	N	Y	N	N	N	Y	Low
Poorolajal (2)	Y	Y	Y	Y	N	N	Y	Y	High
Nejadrahim (3)	Y	Y	Y	N	N	N	N	Y	Medium
Nazmieh (4)	Y	N	Y	Y	N	N	Y	Y	Medium
Vahedi (5)	Y	Y	NO	Y	N	N	N	Y	Medium
Askarian (6)	Y	Y	Y	Y	N	N	Y	Y	High
Ebrahimi (7)	Y	Y	Y	Y	N	N	N	Y	Medium
Aghadoost (8)	Y	Y	Y	Y	N	N	Y	Y	Medium
Lotfi (9)	Y	N	Y	Y	N	N	Y	Y	Medium
Joneidi Jafar (10)	Y	NO	Y	Y	N	N	Y	Y	Medium
Abdi (11)	N	Y	N	N	N	N	Y	N	Low
Rakhshani (12)	Y	Y	Y	Y	N	N	Y	Y	High
Mohammad Nejad (13)	Y	Y	Y	N	N	N	Y	Y	Medium
Azadi (14)	Y	Y	Y	Y	N	N	N	Y	Medium
Kazemi Galougahi (15)	Y	Y	N	Y	N	N	Y	Y	Medium
Gholami (16)	Y	Y	Y	N	N	N	Y	Y	Medium

Mohammadi Nejad (17)	Y	Y	Y	Y	N	N	N	N	Medium
Heidari (18)	Y	N	Y	Y	N	N	Y	Y	Medium
Moradi (19)	N	N	N	Y	N	N	N	N	Low
Nasiri (20)					N	N		Y	Medium
Khalooei (21)					N	N		Y	Medium
Shiva (22)	Y	Y	N	N	N	N	N	NO	Low
Rahnavard (23)					N	N		Y	Medium
Bijani (24)	Y	N	N	N	N	N	N	Y	Low
Mohammadi (25)	Y	Y	Y	Y	N	N	Y	Y	High
Yarmohammadi (26)	Y	Y	NO	Y	N	N	Y	Y	Medium
Ehsani (27)	N	Y	N	N	N	N	Y	N	Low
Farsi(28)	Y	Y	NO	Y	N	N	Y	Y	Medium
Hashemi (29)	Y	Y	Y	Y	N	N	Y	Y	High
Tirgar (30)	Y	Y	N	Y	N	N	Y	Y	Medium
Nejadghaderi (31)	Y	Y	Y	N	N	N	Y	Y	Medium
Ghannad (32)	N	Y	N	N	N	N	N	Y	Low
Sharifian (33)	Y	Y	NO	NO	N	N	Y	NO	Medium
Rezaei (34)	Y	Y	Y	Y	N	N	Y	Y	High
Shoghli (35)	Y	Y	Y	Y	N	N	Y	Y	High
Rezaei (36)	Y	Y	Y	Y	N	N	N	Y	Medium
Adib-Hajbagher (37)	Y	N	Y	Y	N	N	Y	Y	Medium
Bijani (38)	Y	Y	Y	Y	N	N	N	Y	Medium
Gholami (39)	Y	NO	Y	Y	N	N	Y	Y	Medium
Parsa_Pili (40)	Y	Y	Y	Y	N	N	N	Y	Medium
Aghabeigi (41)	Y	Y	N	Y	N	N	Y	Y	Medium
Ghanei Gheshlagh (42)	Y	Y	Y	Y	N	N	Y	Y	High
Lakbala (43)	Y	Y	Y	Y	N	N	N	Y	Medium
Yarahmadi (44)	Y	Y	Y	Y	N	N	Y	Y	High
Mehrdad (45)	Y	Y	Y	Y	N	N	Y	Y	High
Baluchi (46)	Y	Y	Y	Y	N	N	Y	Y	High
Hajivandi (47)	Y	N	N	N	N	N	Y	Y	Low
Izadi (48)	Y	Y	Y	Y	N	N	N	Y	Medium
Mahmoudi (49)	Y	Y	Y	Y	N	N	NO	Y	Medium
Amini (50)	Y	NO	Y	Y	N	N	Y	Y	Medium
Khatony (51)	Y	Y	Y	Y	N	N	N	Y	Medium
Abdifard (52)	Y	Y	Y	Y	N	N	N	Y	Medium
Mohohammadnejad (53)	Y	Y	Y	N	N	N	Y	Y	Medium
Jorvand (54)	Y	Y	Y	N	N	N	N	Y	Medium
Salehifar (55)	Y	Y	Y	NO	N	N	Y	Y	Medium
Javadzadeh (56)	Y	Y	Y	N	N	N	Y	Y	Medium
Geravandi (57)	Y	N	N	N	N	N	N	Y	Low
Jahangiri (58)	Y	Y	Y	Y	N	N	Y	Y	High
Salmanzadeh (59)	Y	Y	Y	N	N	N	N	Y	Medium
Mirzaei-Alavijeh (60)	Y	N	Y	N	N	N	N	N	Low
Moayed (61)	Y	Y	N	Y	N	N	Y	Y	Medium
Rastegari (62)	Y	Y	N	N	N	N	N	NO	Low

Taheri (63)	Y	NO	Y	Y	N	N	Y	Y	Medium
Momen-Heravi (64)	Y	Y	Y	Y	N	N	N	Y	Medium
Ghasemi (65)	Y	Y	Y	Y	N	N	Y	Y	High
Geravandia (66)	Y	N	Y	Y	N	N	Y	Y	Medium
Gharibi (67)	Y	Y	N	Y	N	N	Y	Y	Medium
Joukar (68)	Y	Y	Y	Y	N	N	Y	Y	High
Akbari (69)	Y	Y	Y	N	N	N	Y	Y	Medium
Mehregan (70)	Y	Y	Y	Y	N	N	N	Y	Medium
Abareshi (71)	Y	Y	Y	Y	N	N	N	Y	Medium
Ramezani (72)	NO	N	N	N	N	N	Y	Y	Low
Bagheri Hosseinabadi (73)	Y	Y	Y	Y	N	N	Y	Y	High
Akhuleh (74)	Y	NO	Y	Y	N	N	N	Y	Medium
Fereidouni (75)	Y	Y	Y	NO	N	N	Y	Y	Medium
Sarani (76)	Y	Y	Y	N	N	N	Y	Y	Medium
Siyahkali (77)	Y	Y	Y	Y	N	N	Y	Y	High
Hoboubi (78)	Y	Y	Y	Y	N	N	Y	Y	High
Effatpanah (79)	Y	Y	Y	Y	N	N	Y	Y	High
Rashidi (80)	Y				N	N		Y	Medium
Nouri (81)	Y	Y	Y	Y	N	N	Y	Y	High
Pouryaghoub (82)	Y	Y	Y	Y	N	N	Y	Y	High
Askari Majdabad (83)	Y	Y	N	Y	N	N	N	Y	Medium
Hajimaghsoudi et al(84)	Y	NO	Y	Y	N	N	Y	Y	Medium
Harorani et al(85)	Y	Y	Y	N	N	N	Y	Y	Medium
Sepandi et al(86)	Y	Y	N	Y	N	N	Y	Y	Medium
Roozbeh et al(87)	Y	Y	Y	N	N	N	Y	Y	Medium

Table S1: Quality assignments based on the JBI(Joanna Briggs Institute)

no	Criteria	Yes	No	Not Applicable
1	Were the criteria for inclusion in the sample clearly defined?			
2	Were the study subjects and the setting described in detail?			
3	Was the exposure measured in a valid and reliable way?			
4	Were objective, standard criteria used for measurement of the condition?			
5	Were confounding factors identified?			
6	Were strategies to deal with confounding factors stated?			
7	Were the outcomes measured in a valid and reliable way?			
8	Was appropriate statistical analysis used			

Table S2: JBI quality measurement tools

First author	Year	Sample size	Health worker	Nurse	Prevalence	Quality	Province

Shokouh (1)	2003	88		*	0.33	Low	Tehran
Poorolajal (2)	2004	1000	*		0.24	High	Hamedan
Nejadrahim (3)	2005	434	*		0.53	Medium	West_Azerbaijan
Nazmieh (4)	2005	1020	*		0.39	Medium	Yazd
Vahedi (5)	2006	847	*		0.65	Medium	Kurdestan
Askarian (6)	2007	1555	*		0.50	High	Fars
Ebrahimi (7)	2007	180		*	0.63	Medium	Semnan
Aghadoost (8)	2007	678	*		0.58	Medium	Esfahan
Lotfi (9)	2008	90	*		0.67	Medium	Guilan
Joneidi Jafar (10)	2008	613		*	0.33	Medium	Tehran
Abdi (11)	2009	298	*		0.47	Low	Fars
Rakhshani (12)	2009	231	*		0.65	High	Sistan_Baluchestan
Mohammad Nejad (13)	2009	218		*	0.47	Medium	Tehran
Azadi (14)	2010	111		*	0.46	Medium	Tehran
Kazemi Galougahi (15)	2010	158		*	0.57	Medium	Tehran
Gholami (16)	2010	400	*		0.27	Medium	West_Azerbaijan
Mohammadi Nejad (17)	2010	68		*	0.43	Medium	Tehran
Heidari (18)	2010	77	*		0.74	Medium	Lorestan
Moradi (19)	2010	182	*		0.58	Low	Hamedan
Nasiri (20)	2010	352		*	0.76	Medium	Mazandaran
Khalooei (21)	2010	388		*	0.33	Medium	Kerman
Shiva (22)	2011	355		*	0.49	Low	Tehran
Rahnavard (23)	2011	500		*	0.77	Medium	Guilan
Bijani (24)	2011	172		*	0.32	Low	Qazvin
Mohammadi (25)	2011	138		*	0.53	High	Qazvin
Yarmohammadi (26)	2011	191	*		0.46	Medium	Semnan
Ehsani (27)	2012	328		*	0.45	Low	Tehran
Farsi(28)	2012	200	*		0.57	Medium	Tehran
Hashemi (29)	2012	700	*		0.24	High	Hamedan
Tirgar (30)	2012	340		*	0.60	Medium	Mazandaran
Nejadghaderi (31)	2012	186	*		0.54	Medium	Kerman
Ghannad (32)	2012	89	*		0.52	Low	Hamedan
Sharifian (33)	2012	350		*	0.20	Medium	Tehran
Rezaei (34)	2012	991	*		0.17	High	Tehran
Shoghli (35)	2013	593	*		0.27	High	Zanjan

Rezaei (36)	2013	514	*		0.26	Medium	Tehran
Adib-Hajbagher (37)	2013	298	*		0.38	Medium	Esfahan
Bijani (38)	2013	246		*	0.31	Medium	Qazvin
Gholami (39)	2013	380	*		0.33	Medium	Khorasan Razavi
Parsa_Pili (40)	2013	515	*		0.19	Medium	Tehran
Aghabeigi (41)	2013	385	*		0.76	Medium	Khuzestan
Ghanei Gheshlagh (42)	2014	120		*	0.44	High	Kurdestan
Lakbala (43)	2014	215	*		0.69	Medium	Hormozgan
Yarahmadi (44)	2014	240	*		0.40	High	Tehran
Mehrdad (45)	2014	339		*	0.58	High	Tehran
Baluchi (46)	2015	200		*	0.64	High	Kerman
Hajivandi (47)	2015	68		*	0.59	Low	Bushehr
Izadi (48)	2015	309	*		0.27	Medium	Tehran
Mahmoudi (49)	2015	100		*	0.41	Medium	Tehran
Amini (50)	2015	310			0.68	Medium	Tehran
Khatony (51)	2015	29	*		0.21	Medium	Kermanshah
Abdifard (52)	2015	258	*		0.73	Medium	Kermanshah
Mohohammadnejad (53)	2015	135		*	0.64	Medium	Khuzestan
Jorvand (54)	2015	66	*		0.45	Medium	Ilam
Salehifar (55)	2015	168	*		0.43	Medium	Alborz
Javadzadeh (56)	2015	77		*	0.52	Medium	Esfahan
Geravandi (57)	2016	600	*		0.08	Low	Khuzestan
Jahangiri (58)	2016	168		*	0.54	High	Fars
Salmanzadeh (59)	2016	377	*		0.18	Medium	Khuzestan
Mirzaei-Alavijeh (60)	2014	70		*	0.41	Low	Kermanshah
Moayed (61)	2016	527		*	0.58	Medium	Tehran
Rastegari (62)	2016	430	*		0.34	Low	Khorasan Razavi
Taheri (63)	2016	175		*	0.55	Medium	Esfahan
Momen-Heravi (64)	2016	270	*		0.52	Medium	Esfahan
Ghasemi (65)	2017	267		*	0.41	High	Tehran
Geravandia (66)	2017	600	*		0.13	Medium	Khuzestan
Gharibi (67)	2017	265	*		0.34	Medium	East Azarbaijan
Joukar (68)	2018	1010	*		0.57	High	Guilan
Akbari (69)	2018	343	*		0.28	Medium	Tehran
Mehregan (70)	2018	104	*		0.86	Medium	Khuzestan

Abareshi (71)	2018	223	*		0.33	Medium	Khorasan Razavi
Ramezani (72)	2018	132		*	0.38	Low	Mazandaran
Bagheri Hosseinabadi (73)	2019	616	*		0.25	High	Kerman-Hormozgan-Khorasan Razavi-Mazandaran
Akhuleh (74)	2019	306		*	0.82	Medium	Mazandaran
Fereidouni (75)	2019	176	*		0.51	Medium	Fars
Sarani (76)	2019	142		*	0.52	Medium	Kerman
Siyahkali (77)	2019	420		*	0.13	High	Tehran
Hoboubi (78)	2019	416		*	0.72	High	Khuzestan
Effatpanah (79)	2020	600	*		0.12	High	Khuzestan
Rashidi (80)	2021	380		*	0.54	Medium	Lorestan
Nouri (81)	2021	400		*	0.86	High	Kurdestan
Pouryaghoub (82)	2022	357		*	0.46	High	Tehran
Askari Majdabad (83)	2022	200		*	0.46	Medium	Tehran
Hajimaghsoudi et al(84)	2021	186	*		0.84	Medium	Yazd
Harorani et al(85)	2021	116	*		0.52	Medium	Markazi
Sepandi et al(86)	2023	802	*		0.25	Medium	Tehran
Roozbeh et al(87)	2023	122	*		0.23	Medium	Fars

Table S3 Characteristics of selected studies

1. HOSEINI SS, Ahmadi M. Knowledge and practice of health care workers about needle stick injury and blood borne pathogens in army 505 hospital. 2003.
2. Poorolajal J, Hadadi A, Asasi N, Mohammad K. Frequency of occupational exposure to blood or other potentially infectious materials and related factors in healthcare workers in Hamadan-2003. Iran J Infect Dis. 2004;27:1-9.
3. Nejadrahim R, Gharahughi N, Sistanizade M. Needlestick injuries in the health care workers of Urmia educational hospitals. Nursing And Midwifery Journal. 2005;3(2):0-.
4. Nazmieh H, Najaf-Yarandi A, Janmohammadi S, Hosseini F. ASSESMENT OF THE INJURIES CAUSED BY SHARP INSTRUMENTS IN THE HEALTH WORKERS OF UNIVERSITY HOSPITALS, IN YAZD. Iran Journal of Nursing. 2005;18(43):49-59.
5. Vahedi MS, Ahsan B, Ardalan M, Shahsavari S. Prevalence and Causes of needle stick injuries, in medical personnels of Kurdistan University’s hospitals and dealing with such injuries due to contaminated sharp tools in 1383. Scientific Journal of Kurdistan University of Medical Sciences. 2006;11(2):43-50.
6. Askarian M, Shaghaghian S, McLaws M-L. Needlestick injuries among nurses of Fars province, Iran. Annals of epidemiology. 2007;17(12):988-92.
7. Ebrahimi H, Khosravi A. Needlestick injuries among nurses. Journal of research in health sciences. 2007;7(2):56-62.
8. Aghadoost D, Hajijafari M, Tabatabaei B, Dalirian A. Occupational exposure to blood in the stuff of educational-medical centers of Kashan University of Medical Sciences in 2005. KAUMS Journal (FEYZ). 2007;10(4):59-64.

9. Lotfi R, Gashtasbi A. Needle stick and sharps injuries and its risk factors among health center personnel (astara iran, 2006). Journal of Babol University of Medical Sciences. 2008;10(4):71-7.
10. NA JJ, Shasti M, Izadi M, Ranjbar R, Ghasemi M. Evaluation of frequency of exposure to medical sharp devices among nurses of a university hospital. Journal Mil Med. 2008;10(2):119-28.
11. ABDI MH, Najafipour S, Hamidizadeh S, Jamali F, Pournourouz N. Survey of accidental injuries caused by sharp instruments among the jahrom university of medical sciences hospitals health care workers 2008. 2009.
12. Rakhshani F, Heidari M, Barati S. Prevalence of needlestick injuries among the healthcare professionals in Zahedan medical Sciences university. Iranian Journal of Epidemiology. 2009;4(3):87-91.
13. Mohamadnejad E, Esfandbod M, Ehsani R, Deljo R. Epidemiological aspects of the nurses' occupational exposure to sharp objects. Iranian J Inf Dis. 2009;14(45):47-50.
14. Azadi A, Anoosheh M, Delpisheh A. Frequency and barriers of underreported needlestick injuries amongst Iranian nurses, a questionnaire survey. Journal of clinical nursing. 2011;20(3-4):488-93.
15. Galougahi MHK. Evaluation of needle stick injuries among nurses of Khanevadeh Hospital in Tehran. Iranian journal of nursing and midwifery research. 2010;15(4):172.
16. Gholami A, Salarilak S, Alinia T, Rahim RN. Study of needle stick injuries among health care workers at teaching hospitals in Urmia. Iranian Journal of Epidemiology. 2010;6(3):57-61.
17. Mohammadi Nejad E, Esfand Bod M, Ehsani S, Deljoo R. Needlestick injuries and reporting among emergency nurses in Tehran University of Medical Science. Iranian Journal of Infectious Disease and Tropical Medicine. 2010;48:49-54.
18. Heidari M, Shahbazi S. Prevalence of needle sticks exposure in operation roomâ s staff of Borujen & Lordegan hospitals-2010-2011. Community Health Journal. 2017;5(1):32-7.
19. Moradi A, Mostafavi E, Moradi A. The prevalence and causes of needle stick injuries among the primary health care workers of Bahar city, Hamadan Province. Iran Occupational Health. 2010;7(2):39-42.
20. Nasiri E, Vahedi M, Siamian H, Mortazavi Y, Jafari H. Needle Sticks Injury with Contaminated Blood in the Special Unit, S Staff. Middle-East Journal of Scientific Research. 2010;5(2):61-4.
21. KHALOUEI A, IRANPOUR A, HAMZEHNEZHADI S, RAHMANIAN KE. Study on epidemiology of needle stick injury among nursing personnel of Kerman University hospitals. Kerman, Iran in (2006-2007). 2010.
22. Shiva F, Sanaei A, Shamshiri AR, Ghotbi F. Survey of needle-stick injuries in paediatric health personnel of 5 university hospitals in Tehran. JPMA-Journal of the Pakistan Medical Association. 2011;61(2):127.
23. Rahnavard F, Reza Masouleh S, Seyed Fazelpour SF, Kazemnejad Leili E. Study Factors related to report the needle stick and sharps injuries report by nursing staffs of the Educational and Therapeutic Centers of Guilan University of Medical Sciences, Rasht. Journal of Holistic Nursing And Midwifery. 2011;21(1):30-7.
24. Bijani B, Sotudehmanesh S, Mohammadi N. Epidemiological features of needle stick injuries among nursing staff. 2011.
25. Mohammadi N, Allami A, Mohamadi RM. Percutaneous exposure incidents in nurses: Knowledge, practice and exposure to hepatitis B infection: Percutaneous exposure incidents in nurses. Hepatitis monthly. 2011;11(3):186.
26. Yarmohammadi M. INVESTIGATING THE SEROLOGIC STATUS AND EPIDEMIOLOGICAL ASPECTS OF HEALTH CARE WORKERS'EXPOSURE TO HBV AND HCV VIRUSES. 2011.
27. Ehsani SR, Mohammadnejad E, Hadizadeh MR, Mozaffari J, Ranjbaran S, Deljo R, et al. Epidemiology of needle sticks and sharp injuries among nurses in an Iranian teaching hospital. Archives of Clinical Infectious Diseases. 2012;8(1):27-30.
28. Farsi D, Zare MA, Hassani SA, Abbasi S, Emaminaini A, Hafezimoghadam P, et al. Prevalence of occupational exposure to blood and body secretions and its related effective factors among health care workers of three Emergency Departments in Tehran. Journal of research in medical sciences: the official journal of Isfahan University of Medical Sciences. 2012;17(7):656.
29. Hashemi SH, Torabian S, Mamani M, Moazen Dehkordi S. The Prevalence of Needlestick and Sharps Injuries among Health Care Workers in Hamadan, Iran. Avicenna Journal of Clinical Medicine. 2012;18(4):41-6.
30. Tirgar A, Gholami F. A survey on needlestick injuries and related occupational factors among the nurses. Mod Rehabil. 2012;1:31-8.
31. Nejadghaderi M, Safizadeh H, Khanjani N. The knowledge and practice of medical staff about needle injuries in Rafsanjan's Ali-ebne-Abitaleb hospital, Iran. Health and Development Journal. 2012;1(1):1-10.

32. Ghannad MS, Majzoobi MM, Ghavimi M, Mirzaei M. Needlestick and sharp object injuries among health care workers in Hamadan Province, Iran. Journal of Emergency Nursing. 2012;38(2):171-5.
33. Sharifian S, Aminian O, Afsharisaleh L. Occupational stress and its relationship with need lestick injury among emergency department personnel. 2012.
34. Sh R, Rabirad N, Tamizi Z, Nejad M, Mahmoodi M. Needle sticks injuries among heath care workers in emergency medical centers in Tehran University of Medical Sciences Hospitals (2007-2010). Journal of Health Promotion Management. 2012;1(3):46-54.
35. Shoghli A, MousaviNasab N, Ghorchian F, Masoumi H, Momtazi S. Study of theneedle sticks injury (NSI) among the Zanjan educational hospitals staff. J Adv Med Biomed Res. 2013;21(85):131-41.
36. Rezaei S, RABI RN, Tamizi Z, FALLAHI KM, Nezhad EM. An Investigation into occupational hazards faced by nurses in paediatrics hospitals of Tehran University of Medical Sciences, 2006-2009. 2013.
37. Adib-Hajbaghery M, Lotfi MS. Behavior of healthcare workers after injuries from sharp instruments. Trauma monthly. 2013;18(2):75.
38. Bijani B, Azimiyan J. Epidemiology and risk factors of needle stick injuries among nurses in Bou-Ali Sina teaching hospital, Qazvin. J shahid beheshti nursing and midwifery. 2012;22(78):1-8.
39. Gholami A, Borji A, Lotfabadi P, Asghari A. Risk factors of needlestick and sharps injuries among healthcare workers. International journal of hospital research. 2013;2(1):31-8.
40. PARSA-PILI J, IZADI N, GOLBABAEI F. Factors associated with needle stick and sharp injuries among health care workers. International Journal of Occupational Hygiene. 2013;5(4):191-7.
41. Aghabeigi R, Haghighi S, Asadi M, Adarvishi S, Zadeh M, Ghaderi M. Frequency and factors of injuries by sharp instruments and Needle sticks in operation room's workers in Ahvaz hospitals in 2013. J Clin Nurs Midwifery. 2015;4:1-11.
42. Ghanei Gheshlagh R, Zahednezhad H, Shabani F, Hameh M, Ghahramani M, Farajzadeh M, et al. Needle sticks injuries and its related factors among nurses. Iran Journal of Nursing. 2014;27(89):21-9.
43. Lakbala P, Sobhani G, Lakbala M, Inaloo KD, Mahmoodi H. Sharps injuries in the operating room. Environmental health and preventive medicine. 2014;19(5):348-53.
44. Yarahmadi R, Dizaji RA, Hossieni A, Farshad A, Bakand S. The Prevalence of Needle sticks injuries among health care workers at a hospital in Tehran. Iranian Journal of Health, Safety and Environment. 2014;1(1):23-9.
45. Mehrdad R, Atkins E, Sharifian S, Pouryaghoub G. Psychosocial factors at work and blood-borne exposure among nurses. The international journal of occupational and environmental medicine. 2014;5(1):32.
46. Balouchi A, Shahdadi H, Ahmadidarrehsima S, Rafiemanesh H. The frequency, causes and prevention of needlestick injuries in nurses of Kerman: A cross-sectional study. Journal of clinical and diagnostic research: JCDR. 2015;9(12):DC13.
47. Hajivandi A, Ramavandi B, Rezaeeshiri A, Ahmadi B. A survey on the sharpand cutting wastes injury in nurses of the Bushehr city hospitals in the year 1392. Nursing And Midwifery Journal. 2015;13(6):490-7.
48. Izadi N, Chavoshi F, Sadeghi M. Needlesticks and Sharps injuries among the personnel of Baharlou Hospital in Tehran, Iran. Jundishapur Journal of Health Sciences. 2015;7(4).
49. Mahmoudi N, Sepandi M, Mohammadi AS, Masoumbeigi H. Epidemiological aspects of needle stick injuries among nurses in a military hospital. work. 2015;12(5.64):9-41.
50. Amini M, Behzadnia MJ, Saboori F, Bahadori M, Ravangard R. Needle-stick injuries among healthcare workers in a teaching hospital. Trauma monthly. 2015;20(4).
51. Khatony A, Abdi A, Jafari F, Vafaei K. Prevalence and reporting of needle stick injuries: A survey of surgery team members in Kermanshah University of Medical Sciences in 2012. Global Journal of Health Science. 2016;8(3):245.
52. Abdifard E, Sepahvand E, Aghaei A, Hosseini S, Khachian A. Needle-stick, sharp injuries, and Its related factors among nurses of Imam Reza hospital, Kermanshah, Iran. 2015.
53. Mohammadnejad E, Nemati Dopolani F. Risk factors of needle stick and sharp injuries among health care workers. Journal of Nursing and Midwifery Sciences. 2015;2(1):34-9.
54. Jorvand R, AMIN SF, Ghazanfari Z, SADEGHI RK, Delshad MH. Investigating the factors influencing the behavior of health care workers for needle stick injury (NSI). 2015.
55. Salehifar D, Lotfi R. NEEDLE STICK AND SHARPS INJURIES AND ITS RISK FACTORS AMONG HEALTH CARE PROVIDERS OF A UNIVERSITY HOSPITAL. Iran Occupational Health. 2016;13(2):29-38.

56. Javadzadeh H, Badrian S, Reisi M, Askari N, Meshkati M, Badrian M. A study of the frequency of occupational injuries and knowledge of standard precautions among laboratory staff and nurses. Health Sys Res. 2016;11:671-6.
57. Geravandi S, Alavi SM, Yari AR, Yousefi F, Hosseini SA, Kamaei S, et al. Epidemiological aspects of needle stick injuries among health care workers in Razi Hospital Ahvaz, Iran, in 2015. Archives of Hygiene Sciences. 2016;5(2):85-91.
58. Jahangiri M, Rostamabadi A, Hoboubi N, Tadayon N, Soleimani A. Needle stick injuries and their related safety measures among nurses in a university hospital, Shiraz, Iran. Safety and health at work. 2016;7(1):72-7.
59. Salmanzadeh S, Rahimi Z, Goshtasbipour M, Meripoor M. The prevalence of needle-stick injuries among healthcare workers in Dasht-e-Azadegan, southern west of Iran. International Journal of Pharmaceutical Research and Allied Sciences. 2016;5(2):417-22.
60. Mirzaei-Alavijeh M, Jalilian F, Karami-Matin B, Ghaderi A, Mahboubi M, Janizadeh R, et al. Needle-stick and medication errors in emergency nurses are due to their job stresses? A descriptive study in Kermanshah Hospitals, Iran. J Biol Today's World. 2014;3(3):185-8.
61. Moayed MS, Mahmoudi H, Ebadi A, Nia HS. Stress and fear of exposure to sharps in nurses. Iranian journal of psychiatry and behavioral sciences. 2016;10(3).
62. Rastegari S, Mostafavian Z. " concern of health care workers. the aim of this.
63. Taheri MR, Khorvash F, Hasan Zadeh A. Assessment of mental workload and relationship with needle stick injuries among Isfahan Alzahra hospital nurses. medical journal of mashhad university of medical sciences. 2016;58(10):70-577.
64. Momen-Heravi M, Vakili Z, Telkabadi Z, Soleimani Z. Effective Factors on Needle Stick Injuries in Health Care Workers. International Archives of Health Sciences. 2016;3.
65. Ghasemi M, Khabazkhoob M, Hashemi H, Yekta A, Nabovati P. The incidence of needle stick and sharp injuries and their associations with visual function among hospital nurses. Journal of current ophthalmology. 2017;29(3):214-20.
66. Geravandi S, Moogahi S, Kayedi N, Yari AR, Hedayat M, Shohre S, et al. Investigation of sharp injuries in an educational hospital, Ahvaz, Iran. Archives of Hygiene Sciences. 2017;6(1):10-6.
67. Gharibi F, Asghari-Jafarabadi M, Khodayar Nezhad S, Tajari Z, Esmaeili Z. Study of Needle Stick Injuries Status and its Effective Factors among Nurses. Journal of Research in Applied and Basic Medical Sciences. 2017;3(1):11-9.
68. Joukar F, Mansour-Ghanaei F, Naghipour M, Asgharnezhad M. Needlestick injuries among healthcare workers: Why they do not report their incidence? Iranian journal of nursing and midwifery research. 2018;23(5):382.
69. Akbari H, Ghasemi F, Akbari H, Adibzadeh A. Predicting needlestick and sharps injuries and determining preventive strategies using a Bayesian network approach in Tehran, Iran. Epidemiology and Health. 2018;40.
70. Mehregan N, Adineh M, Saberipour B, Ghorbani P, Hemmatipour A, Alasvand M, et al. The prevalence of sharp object injuries among the operating room staff. Journal of Nursing and Midwifery Sciences. 2018;5(1):25.
71. Abareshi F, Hekmatshoar R, Zokaei M, Akrami R. Survey of occupational exposure to needle stick and its risk factors among Healthcare Workers in one of Sabzevar's hospital. Iran Occupational Health. 2018;14(6):70-7.
72. Etemadinezhad S, Yazdani Charati J. Evaluation of the Prevalence of Needlestick Injuries and Related Factors among Nurses in Sari during 2017. Journal of health research in community. 2018;4(3):34-41.
73. Bagheri Hosseinabadi M, Khanjani N, Etemadinezhad S, Samaei SE, Raadabadi M, Mostafaee M. The associations of workload, individual and organisational factors on nurses' occupational injuries. Journal of clinical nursing. 2019;28(5-6):902-11.
74. Akhuleh OZ, Nasiri E, Heidari M, Bazari Z. Frequency of sharp injuries and its related factors among high-risk wards staff. Journal of Nursing and Midwifery Sciences. 2019;6(4):204.
75. Fereidouni Z, Amirkhani M, Salami J, Najafi Kalyani M. Needle stick and sharps injuries among healthcare workers in Fasa city, Southwestern Iran, 2017. Journal of Occupational Health and Epidemiology. 2019;8(3):156-62.
76. Sarani A, Abdar ZE, Sheikhbardsiri H, Hasani F, Razavi B. Frequency and damage caused by sharp instruments and needle sticks among staff in a university hospital, Kerman, Iran. Journal of Acute Disease. 2019;8(5):200.
77. SIYAHKALI SJM, SEYEDALINAGHI S, Zoha A, SALEHI MR, DADRAS O. Epidemiology of Occupational Injuries in Hospital Personnel: Findings from a Hospital Registry in Tehran, Iran. Journal of International Translational Medicine. 2019;7(1):44-7.
78. Hoboubi N, Asadi N, Ghanavati FK, Jabery O. The association between workload and needlestick injuries among the nurses in the hospitals affiliated with Ahvaz University of medical sciences. Shiraz E-Medical Journal. 2019;20(3).

79. Effatpanah M, Effatpanah H, Geravandi S, Tahery N, Afra A, Yousefi F, et al. The prevalence of nosocomial infection rates and needle sticks injuries at a teaching hospital, during 2013–2014. Clinical Epidemiology and Global Health. 2020;8(3):785-90.
80. Rashidi R, Khoshnamvand M, Mohammadi R, Anbari K. The frequency and causes of occupational injuries caused by Needle Stick among nurses in educational hospitals in Khorramabad in 2020. Yafteh. 2021;22(4):107-19.
81. Nouri S, Tol A, Sadeghi R, Bahmani A, Yaseri M. Predictors of Adherence to Standard Precautions in Preventing Needle Stick Injuries Among the Personnel of Sanandaj Teaching Hospitals, Sanandaj City, Iran. Journal of School of Public Health and Institute of Public Health Research. 2021;18(4):417-34.
82. Pouryaghoub G, Azizi-Alvandi A, Izadi N. Needlestick injury and psychomotor performance as measured by pursuit-aiming test in health-care professionals. Iranian Journal of Nursing and Midwifery Research. 2022;27(2):163.
83. Majdabadi MA, Yazdanirad S, Yarahmadi R, Abolghasemi J, Ebrahimi H. The impact of emotional intelligence and personality traits on the occurrence of unsafe behaviors and needle stick injuries among the nurses. Heliyon. 2022:e09584.
84. Hajimaghsoudi M, Dehghani M, Sadooghian M. Occupational accidents in Yazd University of Medical Sciences hospitals. Occupational Medicine. 2021.
85. Harorani M, Ghaffari K, Jadidi A, Hezave AK, Davodabadi F, Barati N, et al. Adherence to Personal Protective Equipment Against Infectious Diseases Among Healthcare Workers in Arak-Iran. The Open Public Health Journal. 2021;14(1).
86. Sepandi M, Alimohamadi Y, Afrashteh S, Rashti R. Occupational needle stick injuries and related factors among healthcare workers in military hospitals in Tehran. Nursing Open. 2023.
87. Roozbeh J, Malekmakan L, Mashayekh M, Dehghani A, Ansari S, Akbarialiabad H, et al. Exposure to needle stick injuries among health care workers in hemodialysis units in the southwest of Iran: a cross-sectional study. BMC Health Serv Res. 2023;23(1):521.
